# Screening for genital tuberculosis in a limited resource country: case report

**DOI:** 10.1186/s12879-017-2237-8

**Published:** 2017-02-07

**Authors:** Sadie Namani, Emine Qehaja-Buçaj, Diellëza Namani

**Affiliations:** 1 Clinic of Infectious Diseases, University Clinical Center of Kosovo, Prishtinë, Kosovo; 2 Smart Center, Prishtinë, Kosovo; 3 Medical Faculty, University of Prishtina, Kongresi i Manastirit 3A, Prishtinë, Kosovo

**Keywords:** Genital tuberculosis, CA-125, Developing countries, Kosovo

## Abstract

**Background:**

Screening for benign or malignant process of pelvis in young females is a challenge for a physician in a limited resource country. Tuberculosis should be always considered in the differential diagnosis of a pelvic mass in countries with high prevalence of tuberculosis. Negative results of analysis of peritoneal fluid for acid-fast staining, late cultures, and unavailability of new diagnostics methods such as polymerase chain reaction and adenosine deaminase of the aspirated fluid from peritoneal cavity can often result in invasive diagnostic procedures such as laparotomy.

**Case presentation:**

We report a case of a 24 year old Albanian unemployed female living in urban place in Kosovo who presented with abdominal pain, loss of appetite, fever, headache, a weight loss, nonproductive cough and menstrual irregularity for three weeks. In this example case, the patient with cystic mass in tubo-ovarial complex and elevated serum cancer antigen 125 levels was diagnosed for genital tuberculosis after performing laparotomy. Caseose mass found in left tubo-ovarial complex and histopathological examination of biopsied tissue were the fastest diagnostic tools for confirming pelvis TB. The Lowenstein-Jensen cultures were positive after six weeks and her family history was positive for tuberculosis.

**Conclusion:**

Young females with abdominopelvic mass, ascites, a positive family history for tuberculosis and high serum cancer antigen 125, should always raise suspicion of tuberculosis especially in a limited resource country. A laparoscopy combined with peritoneal biopsy should be performed to confirm the diagnosis as this could lead to a prevention of unnecessary laparotomies.

## Background

The incidence of genital tuberculosis (TB) is low and often attacks middle-aged females. However, it is a frequent cause of chronic pelvic inflammatory disease (PID) and infertility in other parts of the world [1]. TB should be always considered in the differential diagnosis of a pelvic mass among immigrants from developing countries, especially those from Asia, the Middle East and Latin America, as well as the human immunodeficiency virus (HIV) positive patients [2]. The most common primary location of genital TB is in Fallopian tubes. The cancer antigen 125 (CA-125) marker is sensitive when used for monitoring the progress of an established tumor [3]. Elevated serum CA-125 is not specific to ovarian cancer, however, and a positive result can, at times, be misleading [2]. In this case report, the elevated serum CA-125, 40 times higher from normal value, was associated with the upper tract genital TB.

## Case presentation

A 24-year-old female patient woman was admitted to our hospital complaining of abdominal pain, loss of appetite, fever, headache, a weight loss, nonproductive cough and menstrual irregularity. Her symptoms started three weeks earlier and she was treated with antibiotics and steroids for Erythema nodosa and Polyarthritis. She had a positive family history for tuberculosis since her father had died two years ago from Pulmonary TB.

At admission, the patient was febrile, intoxicated, hypotensive, anemic, no palpable lymph nodes were noted, normal heart sounds, lungs with rales bilaterally, blood pressure was 90/60 mmHg, respirations 24/min, Pulse 80 beats/min, enlarged liver and spleen, with dolente tenderness in lower left abdominal quadrant, meningeal sings were negative and the rest of physical examinations were normal.

**Fig. 1:**
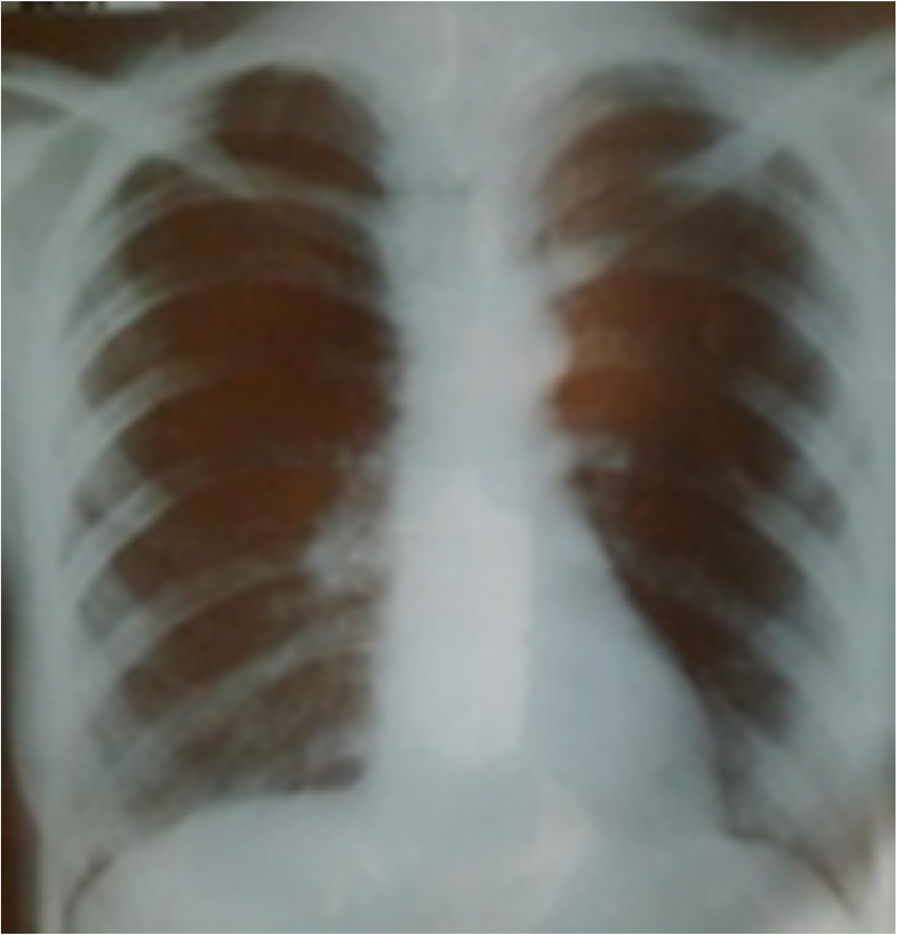
Chest X ray Adenitis hilly bill

**Fig. 2:**
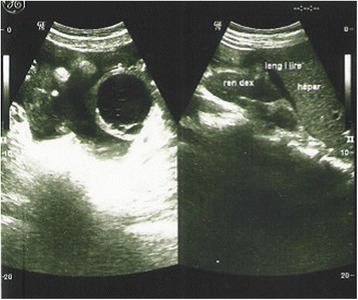
Abdominal ultrasound: cystic formation in left tubo-ovarial complex and ascites

**Fig. 3:**
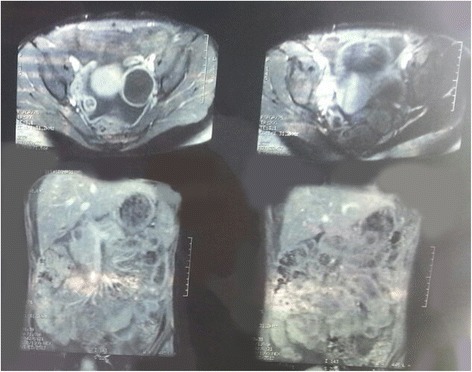
Abdominal MRI: Cystic formation in left tubo-ovarial complex

From laboratory analysis revealed elevated ESR = 40/, HB = 8.7 g/dl, leucocytes = 5.2 × 10^9^/L, CRP = 48, liver enzymes, urea, glucose and kreatinin in normal levels, total proteins = 64 g/L, albumins = 36 g/L, LDH = 492 U/L, Fe = 8.6 mmol/L, blood cultures sterile, Wright negative, Widal negative. Chest x rays bilateral adenitis as shown in Fig.1, Mantoux probe 15 mm, HIV negative, serum CA 125 = 1430…1455, CA 19-9, AFP, CEA and CA 15-3 in normal levels. Ultrasound and MRI of abdomen revealed cystic formation on left tubo-ovarial complex and ascites as shown in Figs. 2 and 3.

Abdominal paracentesis was performed and revealed clear exudative fluid with benign cells. In consultation with gynecologist, the patient underwent operation, suprapubic transversal laparotomy. During operation, in the left tubo-ovarial complex was found caseose mass which was sent together with samples from omentum, peritoneum and ascites for histopathology and microbiological examination. On histopathological examination were seen epiteloidal granulomas with gigantic multinuclear cells, lymphocyte infiltrations and caseous necrosis. On Lowenstein-Jensen cultures was isolated Mycobacterium tuberculosis sensitive to Rifampicin (RIF), Izoniazid (INH), Ethambutol (ETH) and Streptomycin (STM). The patient was treated with a 4-drug TB treatment regimen consisting of RIF, INZ, Pyrazinamide, and ETH for the first two months and with two antituberculotics (RIF and INH) for another 7 months and recovered completely. Her serum cancer antigen 125 level was elevated for three months.

## Discussion

Extrapulmonary tuberculosis is rarely seen at our Infectious Diseases Clinic in Prishtina, with less than 1% (20 cases or 0.6%) of the total 3156 patients hospitalized during 2014. Of the 20 cases treated for extrapulmonary tuberculosis 17 patients were treated for TB Meningitis, and single cases of TB lymphadenitis, TB peritonitis and TB of genital tract.

Abdominal TB, which may involve the genitourinary or gastrointestinal tract, peritoneum, lymph nodes or solid viscera, constitutes up to 12% of extrapulmonary TB and 1–3% of the total, and its nonspecific signs and symptoms may be similar to gastrointestinal (GI) or ovarian cancers [4]. Signs and symptoms of our patients were similar to those observed from previous reports [5–7]. Fever was the main symptom of our patient and it was the most common finding (73%) in the series reported by Muneef et al. [6]. Also, weight loss, menstrual irregularity, ascites, pelvic mass and a positive family history of TB, young age and geographic location in developing countries with high incidence of TB should direct the clinician to the diagnosis of upper genital tract TB. Helpful can be a positive TB skin test which is reported in about a quarter of patients in most reports [7]. Presence of TB at other sites and a positive family history of TB may be helpful in suggesting the diagnosis, but this was reported to occur in somewhat less than 30% of patients [5].

Pelvic TB can be caused by reactivation of the organism (spread via blood stream, lymphatic system or direct from the involved abdominal organs such as intestines) or rarely by venereal transmission [1]. Abdominal ultrasound and MRI of the pelvis was helpful for finding a cystic formation and directing us to suspect of a non-malignant disorder of the genital tract. Non-invasive methods such as tuberculin skin test, chest radiographs or acid-fast staining and culture of the aspirated fluid from peritoneal cavity are usually insufficient to provide the diagnosis of peritoneal or pelvic TB [8, 9]. Abdominal paracentesis revealing clear exudative fluid with benign cells was not helpful, while polymerase chain reaction (PCR) of the aspirated fluid can’t be done in developing countries such as ours. Some recently published studies argue that a positive PCR assay or high adenosine deaminase (ADA) level in aspirated fluid is diagnostic for TB [10, 11]. None of them can be done in our country.

Serum CA-125 is a specific marker for ovarian cancer but can be elevated in various conditions including peritoneal and pelvic TB, endometriosis, pelvic inflammatory disease, liver cirrhosis, chronic renal failure, pleural effusion and pancreas [1, 11–13]. A value of 35 U/mL for CA-125 is considered the upper limit of normal [14]. In this case example, the level of serum CA-125 was 40 times higher from normal value. Cystic formation found by imaging, a positive TB skin test, a positive family history of tuberculosis, abdominal paracentesis were not helpful considering the high level of serum CA-125 found in this case which influenced the decision of the consultant gynecologist for laparotomy instead of laparoscopy. Caseose mass found in left tubo-ovarial complex by suprapubic transversal laparotomy and histopathological examination of biopsied tissue were the fastest diagnostic tools for confirming pelvis TB. The Lowenstein-Jensen cultures of the taken samples confirmed the suspected genital TB although 6 weeks later. For any suspicious mass of the upper genital tract, laparoscopy and histopathological examination of biopsied tissue should be the first intervention to prevent unnecessary surgery and starting appropriate and timely therapy.

## Conclusion

Young females with abdominopelvic mass, ascites, and high serum cancer antigen 125, should always raise suspicion of tuberculosis especially in a limited resource country. A laparoscopy combined with peritoneal biopsy should be performed to confirm the diagnosis as this could lead to a prevention of unnecessary laparotomies.

## Abbreviations

ADA: Adenosine deaminase
AFP: Alpha Fetoprotein
CA-125: Cancer antigen 125
CEA: Carcinoembryonic Antigen
CRP: C reactive protein
ESR: Erythrocyte sedimentation rate
ETH: Ethambutol
GI: Gastrointestinal
HB: Hemoglobin
HIV: human immunodeficiency virus
INH: Izoniazid
LDH: Lactat dehydrogenase
MRI: Magnetic resonance imaging
PCR: Polymerase chain reaction
PID: Pelvic inflammatory disease
RIF: Rifampicin
STM: Streptomycin
TB: Tuberculosis
